# Identifying brain tumor patients’ subtypes based on pre-diagnostic history and clinical characteristics: a pilot hierarchical clustering and association analysis

**DOI:** 10.3389/fonc.2023.1276253

**Published:** 2023-11-29

**Authors:** Simona Esposito, Emilia Ruggiero, Augusto Di Castelnuovo, Simona Costanzo, Marialaura Bonaccio, Francesca Bracone, Vincenzo Esposito, Gualtiero Innocenzi, Sergio Paolini, Chiara Cerletti, Maria Benedetta Donati, Giovanni de Gaetano, Licia Iacoviello, Alessandro Gialluisi

**Affiliations:** ^1^ Department of Epidemiology and Prevention, IRCCS Neuromed, Pozzilli, Italy; ^2^ Mediterranea Cardiocentro, Napoli, Italy; ^3^ Department of Neurosurgery, IRCCS Neuromed, Pozzilli, Italy; ^4^ Libera Università Mediterranea (LUM) “Giuseppe Degennaro”, Casamassima (Bari), Italy; ^5^ Department of Medicine and Surgery, LUM University, Bari, Italy

**Keywords:** central nervous system tumors, cluster analysis, pre-diagnostic history, clinical characteristics, cognitive performance, cancer diagnosis, risk and protective factors, malignancy

## Abstract

**Introduction:**

Central nervous system (CNS) tumors are severe health conditions with increasing incidence in the last years. Different biological, environmental and clinical factors are thought to have an important role in their epidemiology, which however remains unclear.

**Objective:**

The aim of this pilot study was to identify CNS tumor patients’ subtypes based on this information and to test associations with tumor malignancy.

**Methods:**

90 patients with suspected diagnosis of CNS tumor were recruited by the Neurosurgery Unit of IRCCS Neuromed. Patients underwent anamnestic and clinical assessment, to ascertain known or suspected risk factors including lifestyle, socioeconomic, clinical and psychometric characteristics. We applied a hierarchical clustering analysis to these exposures to identify potential groups of patients with a similar risk pattern and tested whether these clusters associated with brain tumor malignancy.

**Results:**

Out of 67 patients with a confirmed CNS tumor diagnosis, we identified 28 non-malignant and 39 malignant tumor cases. These subtypes showed significant differences in terms of gender (with men more frequently presenting a diagnosis of cancer; p = 6.0 ×10^−3^) and yearly household income (with non-malignant tumor patients more frequently earning ≥25k Euros/year; p = 3.4×10^−3^). Cluster analysis revealed the presence of two clusters of patients: one (N=41) with more professionally active, educated, wealthier and healthier patients, and the other one with mostly retired and less healthy men, with a higher frequency of smokers, personal history of cardiovascular disease and cancer familiarity, a mostly sedentary lifestyle and generally lower income, education and cognitive performance. The former cluster showed a protective association with the malignancy of the disease, with a 74 (14-93) % reduction in the prevalent risk of CNS malignant tumors, compared to the other cluster (p=0.026).

**Discussion:**

These preliminary data suggest that patients’ profiling through unsupervised machine learning approaches may somehow help predicting the risk of being affected by a malignant form. If confirmed by further analyses in larger independent cohorts, these findings may be useful to create potential intelligent ranking systems for treatment priority, overcoming the lack of histopathological information and molecular diagnosis of the tumor, which are typically not available until the time of surgery.

## 1 Introduction

Central nervous system (CNS) tumors are quite rare forms of tumors, representing about 1.3% of all cancers. They are hypothesized to have distinct cellular origins, which can be discriminated on the basis of anatomical location, expression of cellular markers, and morphological resemblance to normal brain cells (1). According to the World Health Organization (WHO), there are over 120 different types of brain tumors and data suggest that their incidence is further increasing (2). It is estimated that about 1,000 people receive a new cancer diagnosis every day in Italy (3) and, according to estimates by the National Cancer Registry, approximately 5,700 cases of CNS tumors are diagnosed in the Country each year (4).

CNS tumors are linked with a number of risk and protective factors, including both genetic and environmental factors. The main risk factors include family history of the disease, age, exposure to chemical compounds and radiations (5–7).

Levin and colleagues carried out a large case-control study of more than 400 between cases and controls to investigate whether sensitivity to γ radiation was associated with the risk of CNS tumors (8), and observed that this and the consequent inability to repair DNA damage induced by radiation can increase the risk of such tumors (8). A growing number of studies are supporting the importance of healthy eating in cancer prevention. In particular, a high adherence to Mediterranean Diet (MD) reduces the risk of mortality and the incidence of many types of tumors (7, 9). The protective effects of the MD could be attributed to the high concentration of polyphenols contained in olive oil, wine and vegetables, all foods known for their antioxidant and anti-inflammatory capacity (10, 11). Similarly, omega-3 fatty acids, which are abundant in fish, help slowing down cell proliferation, angiogenesis, inflammation and metastasis (12).

A large number of epidemiological studies have also analyzed the relationship between mobile phone use and the incidence of tumors in the CNS (13, 14), but a meta-analysis of these studies did not reveal any robust statistical evidence for an increase in the risk of malignant or benign neoplasms for a prolonged use of the mobile phone (>10 years) (15). Another potential risk factor is cigarette smoking, which represents a major source of exposure to multiple chemical carcinogens, including polycyclic aromatic hydrocarbons (PAHs) and N-nitroso compounds (16). These cancerous agents are associated with permeability of the blood brain barrier in animal models, along with nicotine (16). As for obesity, the relative risk of all CNS cancers – and especially meningiomas increases with increasing body mass index (BMI) (17).

CNS tumors have also been associated with several socioeconomic factors, occupational and environmental exposures. Inskip et al. found a significant positive association with education and income for low-grade glioma, but not for high grade glioma (18). Among the most reported environmental risk factors were also exposure to agricultural chemicals such as pesticides, insecticides and herbicides (19).

Moreover, studies have indicated that psychological and cognitive manifestations can be considered not only symptoms of CNS tumors but also early warning signs (20, 21), or even risk factors. In fact, a systematic review conducted by Ghandour and colleagues on case reports studies on brain tumors and psychiatric symptoms revealed that in some cases, psychiatric and minor neurological symptoms can emerge even months or years prior to the onset of noticeable neurological signs (22).

Overall, the association of these risk factors with the tumors of CNS has been scarcely investigated, especially through machine learning techniques, which allow to potentially identify subtypes of disease by taking into account also more complex and non-linear relationships among risk factors. This would provide a notable contribution to current knowledge in the field, in light of the modern view that each disease - and even more prominently cancer - has different clinical and biological subtypes, and that each patient is a unique combination of biological, clinical, cultural and psychological characteristics (23, 24).

The aim of this study was to preliminarily investigate the link of different known and suspected risk factors with CNS tumor malignancy, in a cohort of patients elected for neurosurgical treatment. This was accomplished through analysis of associations between diverse exposures which could influence the risk of CNS tumors and their diagnosis - including occupational, socioeconomic, psychometric, nutritional and anthropometric variables, cancer familiarity and history of chronic health conditions - and the different type of tumors, including malignant and non-malignant ones. The very final purpose of this approach is that - shall we identify clusters of patients associated with a higher risk of malignancy - this information may turn useful in future clinical practice, e.g. prioritizing patients for treatment, overcoming the lack of histopathological information and molecular diagnosis of the tumor, which are typically not available until the time of surgery.

## 2 Subjects and methods

### 2.1 Study design

Between October 2018 and March 2020, 90 consecutive patients were enrolled in the MEDICEA (adherence to the MEditerranean DIet in relation to CancEr of brAin) study. Recruited patients (≥ 18 years) had a suspected diagnosis of CNS tumors based on neuroimaging scan and were eligible for surgery at the Neurosurgery Department of the IRCCS Neuromed. Subjects with metastatic and/or recurrent brain tumors were excluded, as well as subjects with confirmed diagnosis of conditions other than brain tumor or with missing diagnosis (see below). Anthropometric measurements and administration of questionnaires were completed before surgery.

The pilot study, conducted according to the principles of the Helsinki declaration, was approved by the Ethical Committee at the IRCCS Neuromed, Pozzilli, Italy (Protocol number: 01262017). All patients signed a written informed consent to be enrolled in the study.

### 2.2 Study population

Trained research personnel from the Department of Epidemiology and Prevention at the IRCCS Neuromed carried out recruitment – carried out between 8.00 and 11.00 a.m. in the Neuromed clinical center and anthropometric measurements, using methods that had been standardized beforehand during preliminary training sessions. Primary CNS tumors were validated through medical records and confirmed by histological reports. Patients without histopathological confirmation or with a diagnosis of brain cysts, secondary tumors or other expansive cerebral processes (n= 22) were excluded. Similarly, one participant who did not complete any questionnaire was filtered out before analysis. Histological information was used to identify main CNS tumors types (i.e. meningiomas 29.5%, glioblastomas 18.2%, adenoma 13.6%, astrocytomas 13.6%, other types 25.1%; **Supplementary Table 1**). Other types of CNS tumors included olygoastrocitoma, chordoma, epidermoid cyst, rolandic tumor, oligodendroglioma, angioma, schwannoma, pituitary adenoma and hemangioblastoma. Additionally, CNS tumors were categorized in malignant (behavior code = 3) and non-malignant (behavior code = 0 or 1) (25).

### 2.3 Definition of variable analyzed

Education was based on the highest qualification attained and was categorized as up to secondary (≤8 y), upper secondary (≥9 y and ≤13 y) and post-secondary (>13 y). Occupational social class was classified as non-manual occupation, manual occupation, retired, housewife and unemployed/unclassified. Marital status was assessed and classified into married, separated/divorced, single and widowed. Household income, expressed as Euros per year, was classified as a four-level variable (<10,000; 10,000-25,000; ≥25,000 Euros/year), with missing values collapsed into a non-respondent category. Smoking status of participants was classified as never-smoker, current smoker or former smoker (i.e. having quitted smoking at least 1 year before enrollment). For clustering purposes, these classes were condensed into never vs ever smokers. Physical activity level was classified into: sedentary, mildly active or physically active lifestyle.

The study sample was also stratified as living in an urban or rural environment on the basis of the urbanization level of the city of residence, as defined by the European Institute of Statistics (EUROSTAT definition) and obtained by the tool “Atlante Statistico dei Comuni” provided by the Italian National Institute of Statistics (www.istat.it) (26).

Height and weight were measured, and BMI was calculated as weight to squared height ratio (kg/m²). Waist circumference was measured according to the National Institutes of Health, Heart, Lung, and Blood Guidelines (27), then waist-to-hip ratio was computed as the ratio between waist and hip, both measured in centimeters. Diastolic and systolic blood pressure were also measured during the visit, through three repeated assessments, and the average values of the last two measurements were taken as the final measure. Diagnosis of hypertension, hypercholesterolemia and diabetes were defined by current pharmacological treatments reported, while history of cardiovascular (angina, stroke and myocardial infarction) and peripheral artery disease was based on self-reported diagnosis.

Patients were also asked about family history of tumor disease within their first-degree family (Yes/No). Furthermore, they were asked whether they lived or worked in proximity of industries, signal relays/repeaters/antennas, sources of asbestos or landfills. The use of mobile phone was also investigated, both asking if patients used to sleep with the mobile phone nearby (Yes/No), and asking how many hours per day they used the phone, with the following potential answers: <2h/day, 2-4h/day and ≥ 4h/day. Finally, patients were asked if they had ever been hospitalized following a head injury due to an accident, a strong bump or a bruise, and if they had undergone previous surgery (Yes/No).

### 2.4 Dietary assessment

Data on food intake during the year before enrolment was collected by the validated Italian version of the EPIC food frequency questionnaire (28) which includes 188 food items, classified into 75 predefined food groups on the basis of similar nutrient characteristics or culinary usage. Adherence to the traditional MD was evaluated by the Mediterranean Diet Score (MDS) developed by Trichopoulou et al. (29) and ranged from 0 to 9 (the latter reflecting maximal adherence).

### 2.5 Psychometric assessment

Quality of life of the patients was assessed through a self-administered Functional Assessment of Cancer Therapy -Brain cancer (FACT-Br) questionnaire before the surgery. This includes five subscales that evaluate physical, social life and family, emotional and functional wellbeing, and additional conditions. The total score ranged from 0 to 184 (the latter indicating higher quality of life) (30).

Psychological resilience was tested in the patients through the Connor-Davidson Resilience Scale (CD-RISC), a self-rated assessment based on 25 items and assessing domains of personal competence, trust/tolerance/strengthening effects of stress, acceptance of change, secure relationships, control, humor, patience, and spiritual influences. Since each item is rated on a 5-point scale (0–4), the total score ranges from 0 to 100, with higher score reflecting greater psychological resilience (31). Global cognitive function was assessed via the Montreal Cognitive Assessment (MoCA). The MoCA is a widely used screening tool that assesses cognitive ability through brief evaluation of various cognitive domains, including visuospatial/executive, naming, memory, attention, language, abstraction, delayed recall and orientation (to time and place) (32). This test incorporates an adjustment for participants with ≤12 years of education, by the addition of 1 point to the final score (33). A total score out of 30 is given, with scores <18 indicating dementia, scores between 18 and 26 indicating mild cognitive impairment and scores ≥26 being classified as cognitively normal. This tool is administered in-person and takes ~10 minutes to complete (33). Depressive symptoms were assessed through the Patient Health Questionnaire 9 (PHQ‐9) self‐administered scale, assessing the nine symptoms most often affected in major depression, namely anhedonia, low mood, alteration of sleeping pattern, altered appetite or eating behavior, feeling of failure/low self-estimate, fatigue, troubles in mental concentration, hypo/hyperactivity behaviors, and suicidal ideation. Each item can receive a score from 0 to 3, depending on how often the relevant domain is affected, with the total PHQ-9 score ranging between 0 (indicating no depressive symptoms at all) to 27 (suggestive of severe depression) (34).

### 2.6 Statistical analysis

Malignant and non-malignant subtypes were compared for a number of variables, which included demographic (age, gender), socioeconomic (education, annual income, occupation), anthropometric (weight, height, BMI, diastolic and systolic blood pressure) and lifestyle variables (smoking habit, physical activity, adherence to MD, daily alcohol and energy intake), as well as psychometric variables (CD-RISC, MoCA, FACT-Br and PHQ-9 scores), professional and other environmental exposures (proximity to industries, exposure to pesticides, insecticides and herbicides). Descriptive analysis of continuous data included the mean and standard deviation (SD) for each group, while the frequency of each class was compared across groups for categorical variables. Fisher Exact tests were applied on the resulting contingency tables for all categorical variables, while unpaired t-test was used for analyzing continuous variables (**Table 1**).

**Table 1:** Characteristics of the sample according to type of central nervous system tumors.

	All CNS (N=67)	Non-malignant CNS (N= 28)	Malignant CNS (N=39)	*p-value*
Gender, men; N (%)	33 (49.2)	8 (28.6)	25 (64.1)	*0.006*
Age, years; mean (SD)	56.3 (14.1)	57.3 (14.7)	56.2 (13.8)	*0.71*
Educational level; N (%)				*0.78*
*Up to secondary*	26 (38.8)	11 (39.3)	15 (38.5)	
*Upper secondary*	24 (35.8)	11 (39.3)	13 (33.3)	
*Post-secondary*	17 (25.4)	6 (21.4)	11 (28.2)	
Occupation; N (%)				*0.88*
*Non-manual*	21 (31.3)	9 (32.1)	12 (30.8)	
*Manual*	10 (14.9)	5 (17.9)	5 (12.8)	
*Retired*	21 (31.3)	9 (32.1)	12 (30.8)	
*Housewife, unemployed and Unclassified*	15 (22.4)	5 (17.9)	10 (25.6)	
Place of residence; N (%)				*0.17*
*Rural*	19 (28.4)	5 (17.9)	14 (35.9)	
*Urban*	48 (71.6)	33 (82.1)	25 (64.1)	
Marital status; N (%)				*0.71*
*Married*	38 (56.7)	15 (53.6)	23(60.0)	
*Divorced/separated*	10 (14.9)	3 (10.7)	7 (17.9)	
*Single*	10 (14.9)	6 (21.5)	4 (10.3)	
*Widowed*	4 (6.0)	2 (7.1)	2 (5.1)	
*Missing*	5 (7.5)	2 (7.1)	3 (7.7)	
Income; N (%)				*0.003*
*< 10,000* Euros/y	11 (16.4)	8 (28.6)	3 (7.7)	
*10,000-25,000* Euros/y	16 (23.9)	10 (35.7)	6 (15.4)	
*≥25,000* Euros/y	24 (35.8)	8 (28.6)	16 (41.0)	
*Non responder*	16 (23.9)	2 (7.1)	14 (35.9)	
Smoking habit; N (%)				*0. 17*
*Never*	36 (53.7)	16 (57.1)	20 (51.3)	
*Ever*	31 (46.3)	12 (42.9)	19 (48.7)	
Diastolic blood pressure, mmHg; mean (SD)	74.1 (19.8)	78.7 (17.5)	70.7 (20.9)	*0.10*
Systolic blood pressure, mmHg; mean (SD)	119.1 (31.9)	124.4 (28.5)	115.4 (33.9)	*0.26*
Body mass index, kg/m^2^_;_ mean (SD)	27.1 (4.8)	27.4 (5.5)	26.9 (4.3)	*0.65*
Waist circumference, cm; mean (SD)	100.0 (14.2)	98.8 (17.0)	100.9 (11.8)	*0.58*
Physical activity level (lifestyle); N (%)				*0.29*
*Sedentary*	40 (59.7)	15 (53.6)	25 (64.1)	
*Mildly active*	15 (22.4)	9 (32.1)	6 (15.4)	
*Physically active*	12 (17.9)	4 (14.3)	8 (20.5)	
Hypertension; N (%)				*0.19*
*No*	39 (58.2)	16 (57.1)	23 (59.0)	
*Yes*	28 (41.8)	12 (42.9)	16 (41.0)	
Diabetes; N (%)				*0.36*
*No*	62 (92.5)	26 (92.9)	36 (92.3)	
*Yes*	5 (7.5)	2 (7.1)	3 (7.7)	
Dyslipidemia; N (%)				*0.14*
*No*	52 (77.6)	19 (67.9)	33 (84.6)	
*Yes*	15 (22.4)	9 (32.1)	6 (15.4)	
Cardiovascular disease; N (%)				*0.82*
*No*	60 (89.5)	25 (89.3)	35 (89.7)	
*Yes*	6 (9.0)	3 (10.7)	3 (7.7)	
*Missing data*	1 (1.5)	0 (0.0)	1 (2.6)	
Cancer familiarity; N (%)				*0.87*
*No*	53 (79.1)	23 (82.1)	30 (76.9)	
*Yes*	12 (17.9)	4 (14.3)	8 (20.5)	
*Missing data*	2 (3.0)	1 (3.6)	1 (2.6)	
Proximity to potential pollution sources; N (%)				*0.13*
*No*	38 (56.7)	14 (50.0)	24 (61.5)	
*Yes*	29 (43.3)	14 (50.0)	15 (38.5)	
Time spent using telephone; N (%)				*0.25*
*<2h/d*	41 (61.2)	18 (64.3)	23 (59.0)	
*2-4h/d*	15 (22.4)	4 (14.3)	11 (28.2)	
*≥4h/d*	9 (13.4)	4 (14.3)	5 (12.8)	
*Missing*	2 (3.0)	2 (7.2)	0 (0.0)	
Sleeping with your phone nearby; N (%)				*0.62*
*No*	34 (50.7)	13 (46.4)	21 (53.8)	
*Yes*	33 (49.3)	15 (53.6)	18 (46.2)	
Old Head Injuries; N (%)				*0.23*
*No*	50 (87.7)	20 (71.4)	30 (76.9)	
*Yes*	7 (12.3)	5 (17.9)	2 (5.1)	
*Missing*		3 (10.7)	7 (18.0)	
Previous surgery; N (%)				*0.11*
*No*	12 (17.9)	3 (10.7)	9 (23.1)	
*Yes*	75 (84.3)	25 (89.3)	30 (76.9)	
Mediterranean Diet Score; mean (SD)	3.5 (1.4)	3.2 (1.1)	3.7 (1.6)	*0.11*
Energy intake, kcal; mean (SD)	2126 (529)	2034 (515)	2191 (536)	*0.23*
Alcohol, g/d; mean (SD)	5.2 (11.7)	5.5 (12.6)	5.0 (11.1)	*0.88*
CD-RISC; mean (SD)	70.1 (13.4)	69.8 (13.4)	70.2 (13.6)	*0.89*
MoCA; mean (SD)	24.0 (3.1)	24.5 (2.9)	23.7 (3.3)	*0.31*
FACT-Br; mean (SD)	146.0 (28.3)	143.3 (28.7)	148.0 (28.2)	*0.51*
PHQ-9; mean (SD)	6.6 (5.7)	6.6 (5.7)	6.6 (5.80.9)	*0.99*

Summary statistics for the total sample analyzed (N=67) and the two degrees of malignancy of brain tumor are reported. P-values (rounded to the second decimal place, unless statistically significant) refer to comparison across these subtypes, which was performed through Fisher Exact Test for categorical variables, and through unpaired t-test for continuous variables.

Statistical association analyses were carried out at the Department of Epidemiology and Prevention of IRCCS Neuromed, through SAS/STAT software, Version 9.4 of the SAS System for Windows©2009.

### 2.7 Hierarchical clustering

Pre-diagnostic history and clinical data also underwent a hierarchical clustering analysis among all the patients with clear diagnosis and definition of malignancy (N=67), in R (37). This analysis, which was aimed at identifying subtypes of brain tumor patients in an agnostic way within the analyzed dataset - based only on anthropometric, socioeconomic, psychometric, lifestyle and other environmental information - was carried out as described in the **Supplementary Methods**, using both a divisive (top-down) and an agglomerative (bottom-up) approach. Briefly, we selected the variables to be included in the analysis, removing collinear features, implemented missing data imputation through a k-nearest neighbor algorithm (see **Supplementary Methods**) and then computed a pairwise (Gower distance) dissimilarity matrix across 67 patients (**Supplementary Figure 1**). Through the Average Silhouette method (**Supplementary Figure 2**), we determined the optimal number of clusters to classify patients based on their clinical and pre-diagnostic characteristics data, then carried out the actual cluster analysis, through which each patient was assigned to one of the clusters. Since divisive clustering has been reported to be more accurate and robust than agglomerative clustering (35) and the two classification methods were significantly homogeneous (Fisher Exact Test p = 0.004; **Supplementary Table 2**; **Supplementary Figure 3**), we took the divisive cluster classification as main exposure analyzed, as in (35). The two resulting clusters of patients, hereafter called Cluster 1 (green, N = 26) and Cluster 2 (red, N = 41), were then compared for all variables mentioned above, through Fisher’s Exact Test (for categorical variables) and through Student’s t test (for continuous variables). Moreover, a Fisher Exact Test was applied to compare the distribution of the two clusters of patients for each subtype of brain tumor identified a priori, and an Odds Ratio with 95% Confidence Interval (OR [CI]) was computed, so to detect potential associations between the two classifications and determine whether the agnostic clustering was somehow reflecting tumor diagnosis.

## 3 Results

**Figure 1:**
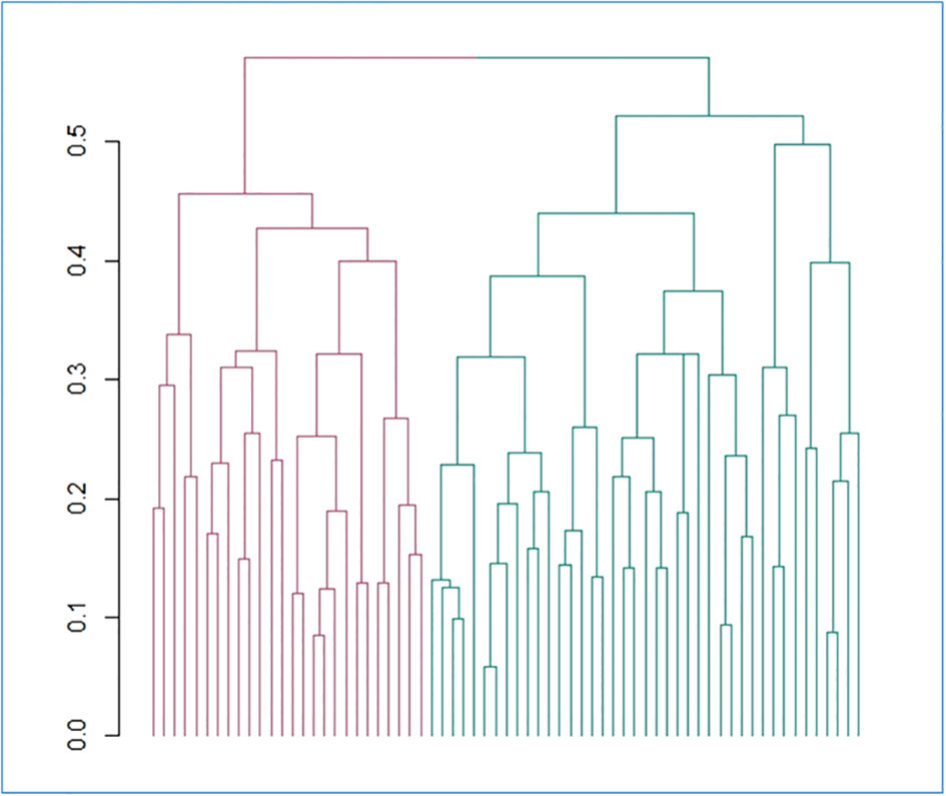
Hierarchical divisive clustering of brain tumor patients, based on the collected features. The dendrogram reporting the clusters identified through divisive hierarchical clustering is reported. Gower distance is reported on the y axis and each single unit analyzed (i.e. patients) on the x axis. Vertical lines correspond to groups (or clusters) of units, while connecting (horizontal) lines identify the distance level at which clusters merge. Legend: red = cluster 1; green = cluster 2.

Basic characteristics of the 67 patients involved in the analyses are reported in **Table 1**. Comparing non-malignant vs malignant CNS tumor cases, we observed a difference in gender distributions across the two groups, with men (representing 49.2% of the total sample) being more prevalent in malignant cases (64.1%), compared to non-malignant ones (28.6%; p = 0.006). Age (mean ± SD = 56.3 ± 14.1 y in the total sample) did not show any difference across the two categories, as educational attainment, occupational class and marital status. However, among socioeconomic and demographic variables, household income showed a differential distribution (Fisher Exact Test p = 0.003), with non-malignant tumors showing the highest percentage of subjects in the average income class (10,000-25,000 Euros; 35.7%), and malignant tumors showing a higher prevalence of people declaring ≥25,000 Euros (41.0%) and presenting many non-responders (35.9%). Hierarchical clustering analysis allowed to compute two clusters of patients based on pre-diagnostic history and clinical data (**Figure 1**), which were compared to analyze their characteristics (**Table 2**). This comparison revealed differences in several characteristics between the two clusters. Patients in Cluster 1 (N=26) were more frequently men (100% vs 17% in Cluster 2; p<0.0001) and smokers (80.8% vs 24.4%; p<0.0001), generally less educated (up to secondary education level: 46.1% vs 34.1%; p = 0.032) and mostly inactive workers (retired: 53.8% vs 17.1%; p<0.0001), with a lower income (≥25.000 Euros/year: 50.0% vs 26.8%; p = 0.002) and a marginal trend toward an older age (mean (SD) age: 60.7(13.3) vs 54.1(14.8); p = 0.061). Likewise, subjects of Cluster 1 reported more frequently a sedentary lifestyle (65.4% vs 56.1%; p=0.049), a previous diagnosis of cardiovascular disease (19.2% vs 2.4%; p=0.018), and a family history of cancer (30.8% vs 9.8%; p=0.012). From a psychometric perspective, Cluster 1 showed worse cognitive performance compared to Cluster 2: (mean (SD) MoCA score: 22.9 (2.4) vs. 24.7(3.3); p=0.023) (**Table 2**). No other difference was detected, except for self-reported proximity to potential pollution sources, such as industries, signal relays/repeaters/antennas, sources of asbestos or landfills (23.1% in Cluster 1 vs 56.1% in Cluster 2; p=0.006. When we compared the classification of tumor cases based on their malignancy vs the agnostic classification of patients made applying hierarchical clustering on pre-diagnostic history and clinical data, we observed an association of Cluster 2 with a lower risk of malignant tumor (OR [95% CI] = 0.26 [0.07-0.86], Fisher Exact Test p = 0.026; **Table 3**).

**Table 2:** Characteristics of the sample according to the two clusters identified.

	Cluster 1 (N=26)	Cluster 2 (N=41)	*p-value*
Gender, men; N (%)	26 (100.0)	7 (17.1)	*<.0001*
Age, years; mean (SD)	60.7 (13.3)	54.1 (14.8)	*0.06*
Educational level; N (%)			*0.032*
* Up to secondary*	12 (46.1)	14 (34.1)	
* Upper secondary*	8 (30.8)	16 (39.0)	
* Post-secondary*	6 (23.1)	11 (26.8)	
Occupation; N (%)			*<.0001*
* Non- manual*	6 (23.1)	15 (36.6)	
* Manual*	4 (15.4)	6 (14.6)	
* Retired*	14 (53.8)	7 (17.1)	
* Housewife, unemployed and unclassified*	2 (7.7)	13 (13.7)	
Place of residence; N (%)			*0.14*
* Rural*	9 (34.6)	10 (24.4)	
* Urban*	17 (65.4)	31 (75.6)	
Marital status; N (%)			*0.004*
* Married*	14 (53.9)	24 (58.5)	
* Divorced/separated*	3 (11.5)	7 (17.1)	
* Single*	5 (19.2)	5 (12.2)	
* Widowed*	1 (3.9)	3 (7.3)	
* Missing*	3 (11.5)	2 (4.9)	
Income; N (%)			*0.002*
* < 10,000* Euros/y	3 (11.5)	8 (19.5)	
* 10,000-25,000* Euros/y	6 (23.1)	10 (24.4)	
* ≥25,000* Euros/y	13 (50.0)	11 (26.8)	
* Non responder*	4 (15.4)	12 (29.3)	
Smoking habit; N (%)			*<.0001*
* Never*	5 (19.2)	31 (75.6)	
* Ever*	21 (80.8)	10 (24.4)	
Diastolic blood pressure, mmHg; mean (SD)	70.2 (23.9)	76.5 (16.6)	*0.20*
Systolic blood pressure, mmHg; mean (SD)	112.3 (39.5)	123.5 (25.5)	*0.16*
Body mass index, kg/m^2^; mean (SD)	28.0 (3.4)	26.6 (5.5)	*0.25*
Waist circumference, cm; mean (SD)	105.1 (10.4)	97.2 (15.3)	*0.046*
Physical activity level (lifestyle); N (%)			*0.049*
* Sedentary*	17 (65.4)	23 (56.1)	
* Mildly active*	5 (19.2)	10 (24.4)	
* Physically active*	4 (15.4)	8 (19.5)	
Hypertension; N (%)			*0.11*
* No*	13 (50.0)	26 (63.4)	
* Yes*	13 (50.0)	15 (36.6)	
Diabetes; N (%)			*0.22*
* No*	23 (88.5)	39 (95.1)	
* Yes*	3 (11.5)	2 (4.9)	
Dyslipidemia; N (%)			*0.14*
* No*	22 (84.6)	30 (73.2)	
* Yes*	4 (15.4)	11 (26.8)	
Cardiovascular disease; N (%)			*0.018*
* No*	21 (80.8)	39 (95.2)	
* Yes*	5 (19.2)	1 (2.4)	
* Missing*	0 (0.0)	1 (2.4)	
Cancer familiarity; N (%)			*0.012*
* No*	17 (65.4)	36 (87.8)	
* Yes*	8 (30.8)	4 (9.8)	
* Missing*	1 (3.8)	1 (2.4)	
Proximity to potential pollution sources; N (%)			*0.006*
* No*	20 (76.9)	18 (43.9)	
* Yes*	6 (23.1)	23 (56.1)	
Time spent using telephone; N (%)			*0.018*
* <2h/d*	16 (61.5)	25 (61.0)	
* 2-4h/d*	7 (26.9)	8 (19.5)	
* >4h/d*	2 (7.7)	7 (17.1)	
* Missing*	1 (3.9)	1 (2.4)	
Sleeping with your phone nearby; N (%)			*0.13*
* No*	15 (57.7)	19 (46.3)	
* Yes*	11 (42.3)	22 (53.7)	
Old Head Injuries; N (%)			*0.059*
* No*	19 (73.1)	31 (75.6)	
* Yes*	2 (7.7)	5 (12.2)	
* Missing*	5 (19.2)	5 (12.2)	
Previous surgery; N (%)			*0.08*
* No*	7 (26.9)	5 (12.2)	
* Yes*	19 (73.1)	36 (87.8)	
Mediterranean Diet Score; mean (SD)	3.9 (1.5)	3.3 (1.3)	*0.09*
Energy intake, kcal; mean (SD)	2234 (588)	2057 (482)	*0.18*
Alcohol, g/d; mean (SD)	7.2 (15.8)	3.9 (8.0)	*0.27*
CD-RISC; mean (SD)	71.5 (14.2)	69.2 (13.0)	*0.50*
MoCA; mean (SD)	22.9 (2.4)	24.7 (3.3)	*0.02*
FACT-Br; mean (SD)	150.2 (29.7)	143.4 (27.4)	*0.34*
PHQ-9; mean (SD)	6.6 (5.7)	6.6 (5.8)	*0.97*

P-values (rounded to the second decimal place, unless statistically significant) refer to the comparison between clusters, which was performed through Fisher Exact Test for categorical variables, and through unpaired t-test for continuous variables.

**Table 3:** Contingency table showing tumor subtype by cluster distribution.

CNS tumors	Cluster 1	Cluster 2
Non-malignant	6	22
Malignant	20	19

## 4 Discussion

**Figure 2:**
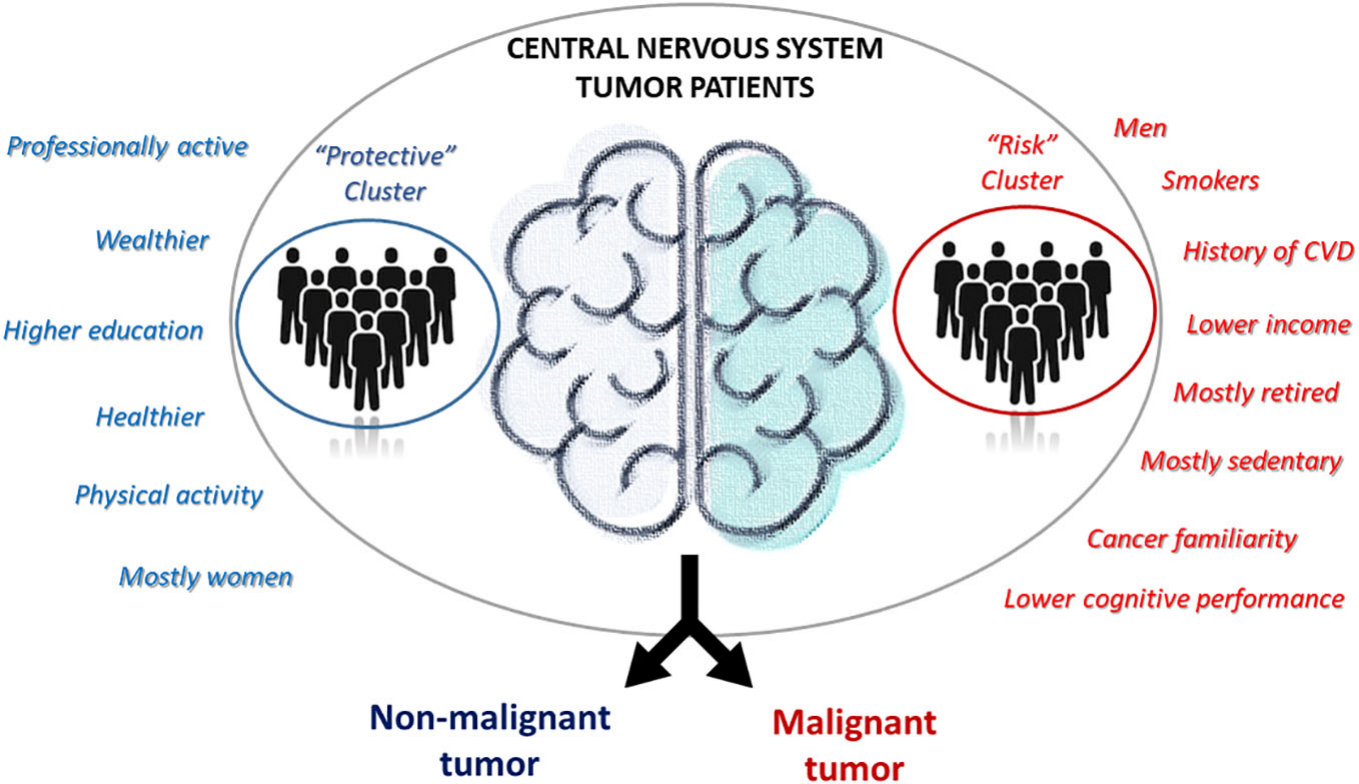
Characteristics of the two patients’ clusters identified.

In this preliminary study, we aimed to investigate the relationship between environmental and biological risk factors and CNS tumor malignancy. We did this through a cross-sectional association analysis between CNS tumor subtypes - divided into non-malignant and malignant CNS tumor cases - and patients clusters derived from a wealth of pre-diagnostic history and clinical data collected within the MEDICEA study. These included not only classical sociodemographic and anthropometric measures, but also environmental exposures, socioeconomic and lifestyle factors, clinical and psychometric features of the patients. We observed two distinct subtypes of patients: one with more professionally active, educated, wealthier and healthier patients, and the other one with mostly retired and less healthy men, with a family history of the disease and lower cognitive performance. Of note, the former cluster showed a protective association with the malignancy of the disease, showing a 74 (14-93) % reduction in the prevalent risk of CNS malignant tumors, compared to the other cluster (**Figure 2**). Since we cannot formally compare our findings with previous evidence due to the lack of studies using a cluster approach to pre-diagnostic history and clinical characteristics of brain cancer patients, we will focus below on the comparison of the evidence derived by this analysis with that produced by classical association studies in the field. Indeed, most of the associations and discrepancies observed between the two risk clusters followed the same trend reported by other studies. As for gender, our results revealed a significantly higher number of men among patients affected by cancer, especially among malignancies, and the totality of our putative risk cluster was made up of men. Previous literature reports a clear predominance of some types of brain tumors in males, such as astrocytomas, glioblastomas multiforme, medulloblastomas, ependymomas and oligodendrogliomas (25), while meningiomas occur more commonly in females than in males, a trend thought to be related to hormonal components (36). Another significant association was detected between self-reported yearly household income and tumor malignancy, with a higher income being associated with the putative risk cluster. Moreover, non-malignant tumors showed the highest percentage of subjects in the average income class (10,000-25,000 Euros/year), while patients with malignant tumors showed a higher prevalence of people declaring ≥25,000 Euros/year and presented many non-responders (37). Part of these non-responders may actually represent people who feel ashamed to self-report a low income (which may actually counteract the imbalance between clusters) and are usually treated as a class. In a study including a total of 11,892 patients with meningiomas, low-grade gliomas, and high-grade gliomas, no clear association was observed between income and the risk of developing brain tumors (38).

While no association was observed between cancer familiarity and tumor malignancy, this was significantly more frequent in the putative risk cluster, in line with previous studies, such as (39), which however reported significant excess of relatedness for astrocytomas, but not for glioblastomas. However, this link is still debated and needs to be clarified, especially with regard to the contribution of shared environmental and genetic factors to the clustering of cases within families (39). Similarly, we observed a higher frequency of ever smokers in the putative risk cluster, in spite of no significant direct association between malignancy and smoking status, but in agreement with previous evidence that active cigarette smoking was associated with an increased risk of CNS tumors in men, but with a reduced risk in women (40), and with gender-differential association between smoking and CNS tumor diagnosis in China (41).

From a more psychological perspective, although none of the psychometric scales assessed revealed direct associations with malignancy degree, the putative risk cluster identified showed a slightly worse cognitive performance. This is in line with the evidence that cognitive deficits are commonly observed in patients with brain tumors (42), and that this could even delay the diagnosis of brain tumor, because these symptoms are often linked to psychiatric diseases (22). However, it remains unclear whether this represents an early marker of brain tumor or a risk factor, a hypothesis which requires long-term longitudinal studies to be tested.

Our pilot study revealed no association with other potential or known risk factors like obesity and use of mobile phone, neither with tumor malignancy nor with risk/protective clusters. While the link with the use of mobile phone is still uncertain and debated (43–45), the lack of associations with obesity is in contrast with previous evidence reported by (17), although this association may be stronger in adolescence, rather than in adulthood (46). Moreover, participants assigned to the putative protective cluster reported more often to live or work in proximity of potential pollution sources like industries, signal relays/repeaters/antennas, sources of asbestos or landfills, which is not in line with a recent review in the field (47). However, this may be partly due to subjects from Cluster 2 more often reporting to live in an urban setting, where there is a higher density of such potential sources of pollution, or simply be a false positive finding.

Overall, we observed here a clear link between patients clinical, lifestyle, psychometric, environmental and socioeconomic profiling and the risk of malignancies, as well as different associations of potential risk factors with the putative risk cluster – in line with previous literature – which we could not always observe when comparing malignant vs non-malignant tumors. This supports the application of machine learning algorithms in stratifying patients based on a combination of risk and protective factors, clinical and biological characteristics, in line with the modern view of cancer epidemiology (23, 24), which represents the essence of personalized medicine and prevention. Should our findings be confirmed by larger independent studies, this information may be useful in the future to create potential intelligent ranking systems for treatment priority, overcoming the lack of histopathological information and molecular diagnosis of the tumor, which are typically not available until the time of surgery. This may ultimately have beneficial implications on timely cancer diagnosis, prognosis and outcomes, possibly increasing survival for patients.

### 4.1 Strengths and limitations

This preliminary study shows some points of strength, but also limitations. Strengths include the originality and novelty of the approach. Although clustering techniques have been already used in brain tumor classification, these were applied to segment brain tumors (48) and identify transcriptomic/immune subtypes useful for prognosis prediction (49) rather than to classify patients’ profiles (49). To our knowledge, the present work is the first report of a cluster analysis based on data other than histological, neuroimaging and molecular characteristics from CNS tumor cases. This may notably improve the power to identify subtypes of disease, by taking into account also potentially complex and non-linear relationships among risk and protective factors. A further novelty consists in comparing CNS tumors based on their malignancy, while they are usually analyzed based on the tissue and cell type affected. The main limitation is represented by the cross-sectional/retrospective approach of the study, due to the current lack of longitudinal prospective data. Indeed, we are still collecting follow-up data after neurosurgery. Also, additional clinical variables like latency, dose-response and tumor localization may have been useful in patients profiling, but were not available at the time of the study due to the limitations imposed to the clinical research activity by the Covid-19 pandemics emergency, which forced us to interrupt recruitment, data collection and assessment. Due to this and to the rarity of the disease, sample size is also relatively small (<100), which may represent a hindrance to statistical power and clustering accuracy. For this reason, these findings warrant further replication in future independent studies on larger sample sizes, possibly including longitudinal data and a wider range of clinical features.

## Author’s note

MEDICEA Study investigators are listed in Supplementary Materials.

## Data availability statement

The datasets presented in this study can be found in online repositories. The names of the repository/repositories can be found below: https://repository.neuromed.it. The password for accessing raw data will be provided upon reasonable request to the corresponding author.

## Ethics statement

The studies involving humans were approved by Ethical Committee at the IRCCS Neuromed, Pozzilli, Italy. The studies were conducted in accordance with the local legislation and institutional requirements. The participants provided their written informed consent to participate in this study.

## Author contributions

SE: Conceptualization, Visualization, Writing – original draft, Data curation, Investigation, Project administration. ER: Conceptualization, Data curation, Formal analysis, Investigation, Project administration, Writing – original draft. ADC: Methodology, Writing – review & editing. SC: Data curation, Writing – review & editing. MB: Funding acquisition, Writing – review & editing. FB: Methodology, Writing – review & editing. VE: Data curation, Project administration, Resources, Writing – review & editing. GI: Data curation, Project administration, Resources, Writing – review & editing. SP: Data curation, Project administration, Resources, Writing – review & editing. CC: Conceptualization, Supervision, Writing – review & editing. MD: Conceptualization, Supervision, Writing – review & editing. GdG: Conceptualization, Supervision, Writing – review & editing. LI: Conceptualization, Funding acquisition, Supervision, Writing – review & editing. AG: Conceptualization, Formal analysis, Visualization, Writing – original draft.
