# Supplementary material for: Identifying brain tumor patients’ subtypes based on pre-diagnostic history and clinical characteristics: a pilot hierarchical clustering and association analysis
Source: Front Oncol. 2023 Nov 29;13:1276253. doi: 10.3389/fonc.2023.1276253 (PMC10749422; doi:10.3389/fonc.2023.1276253)
Supplement: Supplementary file 1 [file DataSheet_1.docx]

***Supplementary Methods***

***Cluster analysis***

The hierarchical cluster analysis was carried out through the following steps:

1. Selection of the variables to include in the study: categorical variables (gender, education level completed, occupation, marital status, income, smoking habit, place of residence, physical activity, sleeping with or using mobile phones more than 3 hours/day, proximity to potential sources of pollution, prevalent hypertension, diabetes, hypercholesterolemia and cardiovascular disease, cancer familiarity) and continuous variables (age, BMI, waist-to-hip ratio, diastolic and systolic blood pressure, daily caloric and alcohol intake, adherence score to Mediterranean diet, psychometric scales assessing depressive symptoms, psychological resilience, cognitive performance and physical, social/family, emotional and functional wellbeing).
2. Removal of collinear variables (defined as those showing absolute value of Spearman rho or Pearson r correlation coefficients >0.6), namely depressive symptoms scale (PHQ9), hypertension, diastolic blood pressure, occupation, marital status and sleeping with mobile phone.
3. Imputation of missing data through a k-Nearest Neighbor algorithm (k=5), implemented through the knn() function of VIM package (<https://cran.r-project.org/web/packages/VIM/index.html>) (1).
4. Computation of the dissimilarity matrix across the 67 patients with confirmed diagnosis of brain tumor subtype, based on pairwise Gower distance. This was computed through the daisy() function of the cluster package (<https://cran.r-project.org/web/packages/cluster/index.html>) (2), applied to the database of clinical and pre-diagnostic history data after imputation and min-max normalization (**Figure S1).**
5. Hierarchical clustering analysis using both a divisive (top-down) and an agglomerative (bottom-up) approach. In divisive hierarchical clustering, all the points in the dataset are initially classified into a single cluster and split is performed recursively as one moves down the hierarchy. In agglomerative clustering, all the elements are initially considered clusters, then the algorithm joins the closest clusters (bottom-up approach) until all the clusters have been merged into a single large cluster containing all the elements. In other words, our analysis clustered subjects based on their degree of pairwise dissimilarity, from highest to lowest (divisive clustering) and from lowest to highest (agglomerative clustering), respectively. This was carried out through applying the hclust() function (cluster package; <https://CRAN.R-project.org/package=cluster>) (3) to the Gower distance matrix computed in the step above.
6. Determination of the appropriate number of clusters (k) to use in assigning patients, based on the Average Silhouette method. This computes the number of clusters which maximizes the average silhouette width, a measure of the quality of clustering indicating how well each object lies within its cluster. This method, applied through the fviz_nbclust() function of the factoextra package (<https://cran.r-project.org/web/packages/factoextra/index.html>) (4), computed two as the optimal number of clusters, which showed the highest average silhouette width (**Figure S2**).
7. Assignment of each patient to one of the two clusters determined above, through application of the cutree() function to the results of the cluster analysis carried out in step 4 (cluster package). This resulted in the two clusters of patients reported in Figure 1 in the main text (divisive clustering) and in **Figure S3** below (agglomerative clustering). The two methods were compared through a Fisher exact test to check for homogeneity of clustering. Despite small discrepancies, classifications of patients through the two different clustering methods were significantly homogeneous (**Table S1**; Fisher Exact Test p = 0.004). In light of this homogeneity and since divisive clustering has been reported to be more accurate and robust, we took the cluster classification resulting from this analysis as main exposure, as in (5).

**Figure S1: Cross-patient dissimilarity matrix based on Gower distance.**

Gower distance (range [0; 1]) represents partial dissimilarities across individuals based on clinical and pre-diagnostic data provided as input. Each squared dot represents a pairwise comparison between patients.

**Figure S2: Computation of the optimal number of clusters (k) to apply in the hierarchical clustering analysis.**


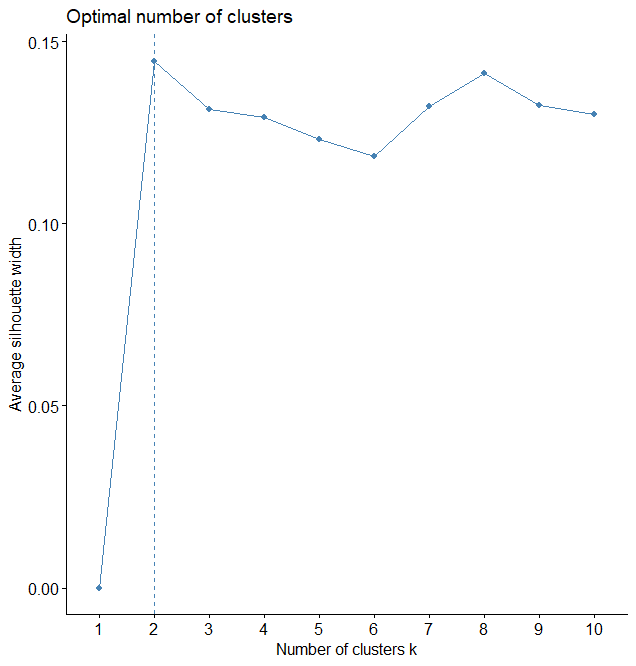


The optimal number of clusters to apply was determined through the Average Silhouette method. The number of clusters (k) is reported on the x axis, while the average silhouette width, a measure of the quality of clustering, indicates how well each object lies within its cluster.

**Figure S3: Hierarchical agglomerative clustering of brain tumor patients.**


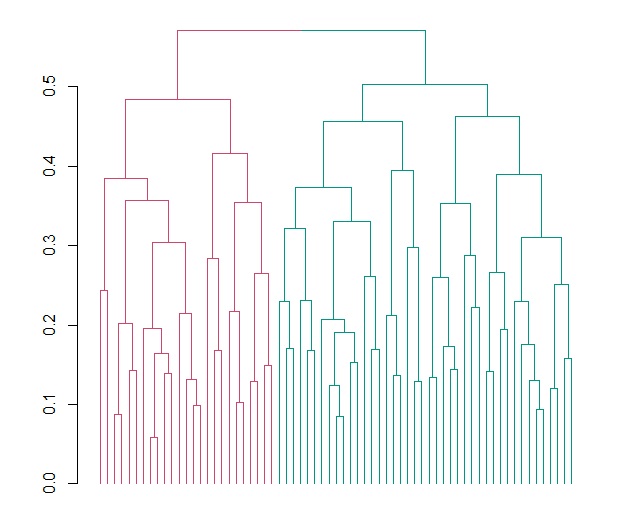


The dendrogram reporting the clusters identified through agglomerative hierarchical clustering is reported. Gower distance is reported on the y axis and each single unit analyzed (i.e. patients) on the x axis. Vertical lines correspond to groups (or clusters) of units, while connecting (horizontal) lines identify the distance level at which clusters merge.

Legend: red = cluster 1; green = cluster 2.

**Table S1: Distribution of CNS tumors diagnoses based on histological characheristics.**

| **CNS tumors types** | **N (%)** |
| --- | --- |
| Meningiomas | 20 (29.5) |
| Glioblastomas | 12 (18.2) |
| Adenoma | 9 (13.6) |
| Astrocytomas | 9 (13.6) |
| Other types | 17 (25.1) |

**Table S2: Contingency table of classifications resulting from Agglomerative vs Divisive Hierarchical clustering.**

| **Hierarchical Clustering Approach** | | **Agglomerative** | |
| --- | --- | --- | --- |
| **Divisive** | **Clusters** | 1 | 2 |
|  | 1 | 22 | 4 |
|  | 2 | 20 | 21 |

**References**

1. Alexander Kowarik, Matthias Templ (2016). Imputation with the R Package VIM. Journal of Statistical Software, 74(7), 1-16. doi:10.18637/jss.v074.i07
2. Maechler, M., Rousseeuw, P., Struyf, A., Hubert, M., Hornik, K. (2019). cluster: Cluster Analysis Basics and Extensions. R package version 2.1.0.
3. Maechler M, Rousseeuw P, Struyf A, Hubert M, Hornik K (2022). cluster: Cluster Analysis Basics and Extensions. R package version 2.1.4 — For new features, see the 'Changelog' file (in the package source), https://CRAN.R-project.org/package=cluster.
4. Alboukadel Kassambara and Fabian Mundt (2020). factoextra: Extract and Visualize the Results of Multivariate Data Analyses. R package version 1.0.7. <https://CRAN.R-project.org/package=factoextra>
5. Di Castelnuovo A, Gialluisi A, Antinori A, Berselli N, Blandi L, Bonaccio M, et al. Disentangling the Association of Hydroxychloroquine Treatment with Mortality in Covid-19 Hospitalized Patients through Hierarchical Clustering. J Healthc Eng. 2021 Jun 25;2021:5556207. doi: 10.1155/2021/5556207.

**Appendix**

**MEDICEA Study Investigators**

**Principal Investigators:** Licia Iacoviello, MD, PhD, (IRCCS Neuromed, Pozzilli and Università dell’Insubria, Varese, Italy), Vincenzo Esposito, MD (IRCCS Neuromed, Pozzilli, Italy), Gualtiero Innocenzi MD (IRCCS Neuromed, Pozzilli, Italy), Sergio Paolini MD (IRCCS Neuromed, Pozzilli, Italy).

**Department of Neurosurgery, IRCCS Neuromed, Pozzilli, Italy:** Vincenzo Esposito, Gualtiero Innocenzi, Sergio Paolini, Grazia Centore, Mariacristina Cotugno, Marianna Damiano, Arianna de Gregorio, Maria Antonietta Di Santo, Cristina Mancarella, Giovanna Santangelo and Piera Sciarra.

**Department of Epidemiology and Prevention, IRCCS Neuromed, Pozzilli, Italy**: Marialaura Bonaccio, Chiara Cerletti, Simona Costanzo, Amalia De Curtis, Giovanni de Gaetano, Maria Benedetta Donati, Simona Esposito, Mariarosaria Persichillo, Alessandro Gialluisi and Emilia Ruggiero.

**Recruitment staff:** Emilia Ruggiero (Coordinator), Mariacristina Cotugno, Simona Esposito and Giovanna Santangelo.

**Data management**: Simona Costanzo, Emilia Ruggiero and Marco Olivieri (Associazione Cuore Sano ONLUS, Campobasso, Italy).

**Data analysis:** Simona Costanzo, Alessandro Gialluisi, Emilia Ruggiero and Augusto Di Castelnuovo (Mediterranea Cardiocentro, Napoli, Italy).

**Biobank and biomedical analyses:** Maria Benedetta Donati, Amalia De Curtis (Coordinator), Emilia Ruggiero, Simona Esposito, Marianna Storto (Department of Analysis Lab Diagnostics, IRCCS Neuromed) and Sara Magnacca (Mediterranea Cardiocentro, Napoli, Italy).

**Informatics:** Marco Olivieri (Associazione Cuore-Sano, Campobasso, Italy).
